# Chlorfenapyr bednets effectively overcome pyrethroid resistance escalation in highly resistant *Anopheles* malaria vectors in Uganda

**DOI:** 10.1038/s41598-025-34493-3

**Published:** 2026-01-02

**Authors:** Ambrose Oruni, Benjamin D. Menze, Yvan G. Fotso-Toguem, Vanessa B. Ngannang-Fezeu, Riccado F. Thiomela, Magellan Tchouakui, Jack Hearn, Jonathan Kayondo, Charles S. Wondji

**Affiliations:** 1 Entomology Department, Uganda Virus Research Institute, P.O. BOX 49, Entebbe, Uganda; 2 Centre for Research in Infectious Diseases, LSTM-Research Unit, P.O BOX 3591, Yaoundé, Cameroon; 3 Liverpool School of Tropical Medicine, Vector Biology Department, Liverpool, L3 5QA UK; 4 Centre of Epidemiology and Planetary Health, School of Veterinary Medicine, Scotland’s Rural College, Inverness, UK; 5 International Institute of Tropical Agriculture (IITA), P.O. Box 2008, Yaoundé, Cameroon

**Keywords:** Experimental huts, New generation-LLINs, Interceptor G2, Malaria vectors, *Anopheles funestus*, *Anopheles gambiae*, Resistance escalation, Uganda, Diseases, Ecology, Ecology, Zoology

## Abstract

Escalating insecticide resistance threatens the efficacy of LLINs, undermining malaria control in Africa. We conducted the first experimental hut trials in Uganda using highly resistant free-flying wild *Anopheles* mosquitoes and F2 hybrids of FANG and Uganda *An. funestus* to evaluate the performance of bednets. The interceptor G2 (chlorfenapyr) bednet demonstrated superior efficacy compared to Interceptor (pyrethroid-only) net [mortality odds ratio (OR): 18.7 (8.05–48.6) *P* < 0.0001], achieving an overall mortality rate of 70.6% and 63.2% against *An. funestus* and *An. gambiae* respectively. In contrast, PermaNet 3.0 and Olyset Plus (piperonyl butoxide (PBO)) and Royal Guard (pyriproxyfen (PPF)-treated) bednets exhibited significantly lower mortality against *An. funestus* [Olyset Plus: 36.1%, PermaNet 3.0: 31.0% and Royal Guard (37.6%], though performance against *An. gambiae* was moderate [PermaNet 3.0: 61.4%, Olyset Plus: 50.0%, Royal Guard: 51.6%]. Interceptor net produced the lowest mortality (~ 25%) against both species. Regarding blood-feeding inhibition (BFI), PBO nets, particularly Olyset Plus, outperformed Interceptor G2 and Royal Guard, while Interceptor produced minimal BFI (< 36%). Further evaluation of Royal Guard’s PPF effect on oviposition revealed no significant reduction in oviposition rates compared to controls with *An. funestus* (63.9% vs. 63.3%, *P* > 0.05). Genetic analysis using the hybrid crosses revealed that pyrethroid resistance markers (*4.3 Kb-SV* and *G454A-Cyp9K1*) were significantly associated with mosquito survival and blood-feeding success against PermaNet 2.0 (pyrethroid-only) and PermaNet 3.0 but showed no significant association with Interceptor G2 net. These findings support Interceptor G2 as a promising intervention for regions dominated by both highly resistant *An. funestus* s.l. and *An. gambiae* s.l. Piperonyl butoxide and PPF nets emerge as a good alternative for areas mostly dominated by resistant *An. gambiae* s.l. populations. Critically, the demonstrated variable impact of insecticide resistance on bednet efficacy underscores the imperative need for a comprehensive vector distribution mapping, continuous field efficacy assessments, and systematic resistance monitoring. This evidence-based triad should guide strategic LLIN distribution and rotations to sustain malaria control efficacy in resistance-prone settings.

**Supplementary Information:**

The online version contains supplementary material available at 10.1038/s41598-025-34493-3.

## Introduction

Sub-Saharan Africa continues to face a high malaria burden despite widespread vector control efforts, particularly through bednets, with progress stalling in recent years^1^. Insecticide-based interventions, primarily long-lasting insecticidal nets (LLINs) and indoor residual spraying (IRS), remain the cornerstone of malaria control^1^. LLINs have been the primary malaria control tool in endemic countries, due to their ease of deployment, cost-effectiveness, and ability to offer both personal and community protection^2^. Until recently, pyrethroids were the sole insecticide class used in LLINs. However, rising pyrethroid resistance across Africa has significantly reduced the efficacy of pyrethroid-only LLINs, leading to the development of piperonyl butoxide (PBO) LLINs and subsequently other dual-active-ingredient (ai) bednets. The synergist PBO enhances pyrethroid potency by inhibiting cytochrome P450 oxidases (CYPs), key drivers of insecticide resistance in major malaria vectors^3,4^. In principle, PBO-treated LLINs are expected to exhibit greater insecticidal efficacy than pyrethroid-only LLINs, a finding that has been consistently demonstrated in laboratory studies^5–8^. Following a cluster-randomized trial in Tanzania^9^, the WHO recommended PBO LLINs, prompting their rollout in endemic regions with the target of achieving universal coverage.

In Uganda, large-scale LLIN distribution began in 2013–14, with pyrethroid-only nets; Olyset net, and PermaNet 2.0, and then every three years as per WHO guidelines^10^. In 2017, PBO LLINs were progressively introduced in western and eastern districts during the 2017–18 campaign, integrated into a trial across 104 health sub-districts^11^. Clinical and entomological results from areas that received PBO LLINs compared to pyrethroid-only nets showed lower parasite prevalence and vector density^12,13^, aligning with a Cochrane review^14^. Unfortunately, while early semi-field studies (pre-2015) confirmed PBO LLINs’ high efficacy^15–17^, recent trials indicate declining performance, with lower mortality rates against *An. gambiae* in Benin^18,19^ and *An. funestus* in Cameroon^5^. However, there is a vast variation in the performance of PBO LLINs in different localities which could be attributable to vector behaviours and differences in insecticide resistance profiles.

To counter ongoing resistance threats, new insecticide classes — pyrroles and neonicotinoids — were introduced in 2018 following WHO prequalification^20^. A pyrrole, chlorfenapyr, is combined with pyrethroids in “new-generation” dual-AI nets like Interceptor® G2. Chlorfenapyr disrupts the energy production in insects, leading to their death^21,22^. The recent study published by Tchouakui et al revealed that the primary malaria vectors remain susceptible to pyrroles^23^. Furthermore, semi-field studies highlight Interceptor® G2’s superior efficacy against resistant wild vectors compared to other LLINs^24–26^. In Uganda, chlorfenapyr-treated nets; Interceptor® G2 and PermaNet®Dual were rolled out in November 2023, though its performance remains unevaluated locally. However, trials in Tanzania’s Lake Victoria basin, showed promising results revealing high efficacy^26,27^. Another dual-AI net, Royal Guard®, combining pyrethroids with pyriproxyfen (PPF) — an insect growth regulator—was distributed in mostly northern and eastern districts of Uganda in 2020^28^. This type of LLIN offers both high mortality and reduces vector oviposition and fecundity^29^. The entomological impact of PPF-LLINs in Uganda is untested, but epidemiological data suggest performance comparable to PBO nets^28^. However, trials in Tanzania reported only moderate efficacy against malaria vectors, with performance declining over time^27^.

In eastern Uganda, two major malaria vectors; *An. funestus* s.l. (referred to here as *An. funestus*) and *An. gambiae* s.l. (referred to here as *An. gambiae*) dominate^8,30^ although their relative abundance affected by ongoing control interventions^31–35^. In Mayuge district where the trial was conducted, we previously showed that *An. funestus* is the dominant vector and both vectors exhibit extreme pyrethroid resistance, up to ten times the diagnostic dose^36,37^, threatening LLIN efficacy^5,36–38^. Here, we conducted the first experimental hut trial in Uganda, in a district along the Lake Victoria basin. Following WHO guidelines^39^, the study simultaneously evaluated the performance of five types of LLINs, including both conventional and next-generation bednets, against these highly resistant vectors.

## Methods

### Study area

**Fig. 1:**
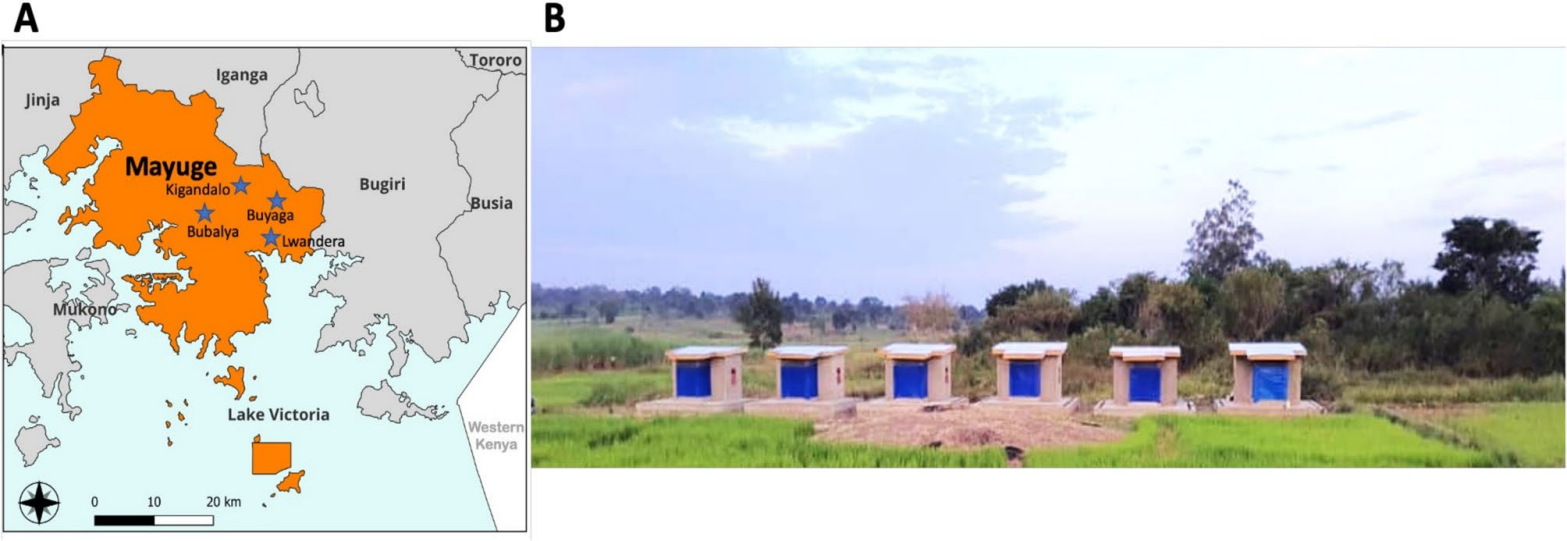
Location of Mayuge and picture of the CRID-UVRI newly constructed experimental huts. (**A**) Map showing the location of Kigandalo village (yellow arrow) in Mayuge district. (**B**) Picture of the newly constructed West African style experimental huts in Uganda.

The study was conducted in the eastern region of Uganda in the district of Mayuge (0°23′24.3"N 33°37′36.0"E) (Fig. 1A). The district is along the shores of Lake Victoria in the south and has a tropical savanna climate where the average temperature is 26 °C and rainfall is received throughout the year with an average of 131 mm. The main economic activities include fishing and farming of cash crops such as cotton, coffee, sugar cane and rice. Eastern Uganda has one of the highest malaria transmission rates in the country and Mayuge district belongs to areas categorised as “very high transmission” by the National Malaria Control Program (NMCP) and was among the first districts to receive LLINs in 2012^40^. Although malaria transmission occurs typically throughout the year, it is largely perennial with two annual peaks following two rainy seasons (March–May and August to October).

The experimental hut trial was conducted in Kigandalo sub-county, Kigandalo parish, Kigandalo B village (0°23′24.3"N 33°37′36.0"E) where a total of six (6) huts were constructed (Fig. 1B) according to WHO standard^39^ . The site is surrounded by permanent water sources, rice fields and sugar cane plantations with permanent flowing streams nearby and a low water table which is suitable for *An. funestus* breeding as well as *An. gambiae*. The main malaria vector species are *An. funestus* and *An. gambiae* with the former being the predominant vector. Other mosquitoes such as *Culex* and *Mansonia* species are also present with the latter being very scarce^36,37^. Both malaria vector species are very resistant to insecticides especially pyrethroids even at higher concentrations (5 × and 10 × the diagnostic dose) but susceptible to organophosphates, neonicotinoids, and pyrroles^36,37,41,42^. The experimental hut effect test and trial were conducted against free-flying mosquitoes for a combined 9 weeks between 26^th^ August 2023 to 18^th^ November 2023 during part of the dry season and most of the second rainy season.

### Experimental hut design

We opted for the West African design purely for logistical reasons because they were easiest and cheapest to build, which is well-suited to the goal of the study. These experimental huts were constructed following the WHO guidelines (WHO, 2016b). The entire structure is built using cement, sand, and bricks with plastered walls. The roof is built with corrugated iron sheets with a ceiling made of plywood. Four windows; two in front and two on each side are designed with 2 cm angular gap which allows mosquitoes to enter but not escape the huts. A veranda trap is added to the design at the back of the hut and built according to the WHO guidelines (WHO, 2016b). To separate the veranda from the rest of the room, a curtain is used. Before sleeping, each volunteer is required to raise the curtain to give mosquitoes an opportunity to exit the room to the veranda. The curtains are lowered in the morning before mosquito collections begin in the room to prevent exited mosquitoes from coming back into the room hence separating the veranda collections from room/hut collections.

### Net treatment arms and comparison

During the trial, five (5) types of LLINs plus a control were assessed which comprised of one pyrethroid-only net, four next-generation bednets and one untreated net as control (Table 1). As per the WHO protocol, square holes measuring 4 cm x 4 cm were cut into bednets, two on each of the long sides and one on each of the short sides.

**Table 1:** Characteristics of bednets tested in the experimental huts.

Treatment arm	Holding time	Description	Manufacturer
Untreated (Control)	24 h—72 h	100% polyester with no insecticide	Local Market
Interceptor	24 h	200 mg/m^2^ of alpha-cypermethrin impregnated in polyester material	BASF Agricultural Solutions
Interceptor G2 (IG2)	72 h	200 mg/m^2^ chlorfenapyr and 100 mg/m^2^ alpha-cypermethrin impregnated in polyester and polyethylene material	BASF Agricultural Solutions
Royal Guard (RG)	24 h	208 mg/m^2^ of alpha-cypermethrin + 208 mg/m^2^ of Pyriproxyfen impregnated in polyethylene material	Disease Control Technologies (DCT)
Olyset Plus	24 h	800 mg/m^2^ of Permethrin + 400 mg/m^2^ PBO impregnated in polyethylene material	Sumitomo Chemical
PermaNet 3.0	24 h	84 mg/ m^2^ of deltamethrin on the sides and 120 mg/m^2^ on the top + 800 mg/m^2^ PBO on the top impregnated in polyester material	Vestergaard

### Hut effect test

Prior to the study, the hut effect was assessed between 26^th^ August and 16^th^ September 2023 to evaluate if the huts and/or the participants were individually attractive. During these 3 weeks, volunteers slept under the untreated nets hanging in the six newly constructed huts and collected mosquitoes every morning.

### Experimental hut trial

The experimental hut trial was carried out following a 36-nights protocol as described by WHO guidelines for laboratory and field-testing of long-lasting insecticidal nets^43^. To correct any specific attractiveness observed during the hut effect assessment, the Latin square design rotation was used to alternate bednets^43^ such that at the end of the experiment, each bednet would have spent six days in each hut. The huts were cleaned at the end of the week before the next net rotation. Six adult male volunteers were selected to sleep in each hut from 20:00 GMT in the evening to 5:00 GMT in the morning. Each day, the sleepers were rotated such that at the end of the week, a sleeper would have spent one night in each hut. The sleeper rotation was done to correct any bias that could be because of specific attractiveness from the sleepers. Sleepers were blinded to treatment allocation by concealing the net type and covering all visible or readable labels to prevent identification.

### Mosquito collections

Every morning, alive and dead mosquitoes were collected using haemolysis tubes^5^ from: (i) inside the bednets (ii) from the sleeping area (room); on the floor, walls, and ceiling, and (iii) in the veranda exit trap. The mosquitoes collected from each compartment were kept in well labelled separate bags to avoid mixing any samples from the different compartments. The collected samples were then categorized as dead, alive, blood fed or unfed. The ‘alive’ mosquitoes were transferred into paper cups and fed with 10% sugar solution and the mortalities were recorded at 24 h for Interceptor, PermaNet 3.0, Olyset Plus and RG bednets and 72 h for IG2. Furthermore, for RG net and control, alive and blood fed mosquitoes were kept for 4 days and then forced to lay eggs^44^ to compare the oviposition rates. The collected females were sorted according to morphological keys as previously described^45^.

### Assessing performance of the bednets

Performance of the bednets were assessed relative to the control net (untreated net) as described by WHO^43^ in terms of:

- Deterrence rate: the decrease in the proportion of mosquitoes entering the hut as compared to the control. Deterrence (%) = 100 × (Du − Dt)/Du, where Du is the total number of mosquitoes collected in the control hut and Dt is the total number of mosquitoes collected in the treated hut.
- Entry rate: the proportion of mosquitoes found to have successfully entered the hut. Entry rate (%) = 100 × (Ht/Hn) where Ht is the total number of mosquitoes found in each hut and Hn is the total number of mosquitoes collected in all the other five treated huts.
- Exophily (exit rate): the proportion of mosquitoes found to have exited to the veranda trap in each of the huts. Exophily (%) = 100 × (Ev/Et) where Ev is the total number of mosquitoes found in veranda and Et is the total number of mosquitoes found both inside the hut and veranda. A component of an exit rate is Induced exophily which is the proportion of mosquitoes that exited to the veranda trap because of the presence of a treated net. Induced exophily (%) = 100 x (Et-Ec)/(100-Ec) where Et is exophily rate in hut with treated net and Ec is the exophily rate in hut with control net.
- Blood feeding rate (BFR): the proportion of mosquitoes found to have successfully taken a blood meal. Blood feeding rate = (N mosquitoes fed) × 100/total N mosquitoes. Where “N mosquitoes fed” is the number of mosquitoes that have blood fed, and “total N mosquitoes” is the total number of mosquitoes collected.
- Blood-feeding inhibition (BFI): the reduction in blood-feeding in comparison with the control hut. BFI is calculated as; (1-Bt/Bc*100) where Bt is the BFR of mosquitoes in hut with the treated net and Bc is the BFR of mosquitoes in hut with the control net. BFI is an indicator of personal protection (PP). Specifically, the PP effect of each bednet is the reduction in the rate of blood feeding induced by the net when compared to the control. Personal protection (%) = 100 × (Bu-Bt)/Bu, where Bu is the total number of blood fed mosquitoes in the control and Bt is the total number of blood-fed mosquitoes in the treated hut.
- Immediate and delayed mortality: immediate mortality is the proportion of mosquitoes found dead in the morning while delayed mortality is the proportion of alive mosquitoes that die after 24 h to 72 h with access to sugar solution. This study focused on the overall mortality (immediate plus delayed mortality) and is calculated as: Mortality (%) = 100 × (Mt/MT) where Mt is the total number of mosquitoes found dead in the hut even after the holding period and MT is the total number of mosquitoes collected in the hut. Mortality is also an indicator of the killing effect of a bednet which is calculated as; Killing effect (%) = 100 x (Kt – Ku) / Tu, where Kt is the number of mosquitoes killed in the hut with the treated net, Ku is the number of mosquitoes killed in the hut with untreated net and Tu is the total number of mosquitoes collected from the hut with untreated net. For superiority trials we determine whether there is a significant difference (at the 95% significance level) between mortality in the two arms of the trial being considered.

### Bednet superiority assessment

The methodology used to assess the superiority of ITNs was proposed by WHO^46^. A superiority trial could be performed to show if a particular insecticide-treated bednet is superior to another bednet. In this study, we assessed if PPF net (Royal Guard), PBO nets (PermaNet 3.0 and Olyset Plus) and chlorfenapyr net (Interceptor G2) were superior to a pyrethroid-only net (Interceptor). Three parameters; mortality, blood feeding rate and exit rates were considered and the assessment of whether a bednet is superior was made by determining if there is a significant difference (at the 95% significance level) between a treated bednet and untreated net. We further calculated the Odds ratio (OR) produced by each bednet in comparison to Interceptor G2. The analysis was performed in R software as described by Challenger et al.^47^. Briefly, a regression model is fitted, grouping data of same type of bednets together and the OR with its confidence interval (CI) constructed estimated regression parameters. A bednet is considered superior when there is a statistically significant difference in the measured parameter between the treated and untreated bednets.

### Assessment of impact of 4.3 Kb-SV and AG454A-Cyp9K1 resistance markers on bednet performance against An. funestus mosquitoes

To assess the impact of resistance markers on the efficacy of bednets, we conducted a second experimental hut trial using F₂ hybrids derived from field-collected *An. funestus* mosquitoes from Mayuge crossed with the FANG laboratory-susceptible strain, which is maintained at the Centre for Research in Infectious Diseases (CRID) in Cameroon. The use of hybrid mosquitoes was intended to obtain the segregated genotypes of two key resistance markers, *4.3 Kb-SV* and *G454A-Cyp9K1*, which are already fixed in Ugandan populations (only RR genotype present). This approach allowed for the segregation of genotypes, enabling robust genotype–phenotype association analyses as successfully done in the past^48–50^. These two markers were selected as proxies for pyrethroid resistance, as they have been previously validated to be strongly associated with resistance phenotypes^51,52^.

Field mosquitoes collected in November 2024 were reared to F_1_ progeny, then crossed with the FANG strain to generate F_2_ hybrids. The experimental hut trial was conducted over three nights using a release-recapture protocol that featured two key modifications: (1) windows were sealed internally to prevent mosquito escape, and (2) 75–100 mosquitoes were released per hut. Six treatments were evaluated (i) Untreated net, (ii) PermaNet 3.0, (iii) Olyset Plus, (iv) PermaNet Dual, (v) Interceptor G2 and (vi) PermaNet 2.0.

In this study, we assessed impact of resistance on collected mosquitoes from PermaNet 3.0 (representing PBO nets), Interceptor G2 (representing chlorfenapyr nets), and PermaNet 2.0 (representing pyrethroid-only net). The alleles of resistance markers were genotyped in a subset of these treatments to determine the impact of pyrethroid resistance on the performance of the bednets. The following phenotypes were included: the dead, alive, blood-fed, and unfed mosquitoes in the veranda, inside the net and in the room. However, only two LLIN performance parameters were considered; mortality and blood feeding status, since enough samples could be obtained. The association between the resistance and the performance of each net was assessed using ‘vcd’^53^and ‘epitools’^54^ packages in R software, to calculate the Fisher’s exact probability test using a contingency table.

### Data analysis

The number of mosquitoes collected in each hut with different treatments were assessed using negative binomial regression. The proportions of entomological outcomes assessing performance of bednets such as blood feeding, exophily, mortality, for a given experimental hut treatment were assessed using binomial generalized linear mixed models (GLMMs) with a logit link function, fitted using the ‘*lme4*’ package^55^ for R software (version 4.2.2). An independent model was fitted for each entomological outcome and mosquito species. To complement the fixed effect of each treatment, random effects were included in each model to account for these sources of variation; between the six huts, between the six sleepers, between the six weeks of the trial. Hut trial data was analysed using experimental hut trial data analysis pipeline developed by Challenger *et al*^47^. Finally, for the Hut effect test, the one-way analysis of variance (ANOVA) was used to determine if there was a significant difference in the means of mosquitoes collected from the six huts. For the statistical analyses in this study, level of significance (alpha) was set at a cut-off value of 0.05 and correction was done for multiple testing. The relationship between bednet performance and resistance marker genotypes was evaluated by conducting a phenotype-genotype association analysis, examining mortality and blood feeding rates using Unconditional Maximum Likelihood Estimation (MLE) and Wald normal approximation method in R software using packages ‘*epitools*’^54^ and ‘*oddsratio*’^56^.

## Results

### Hut effect test and overall mosquito abundance

**Fig. 2:**
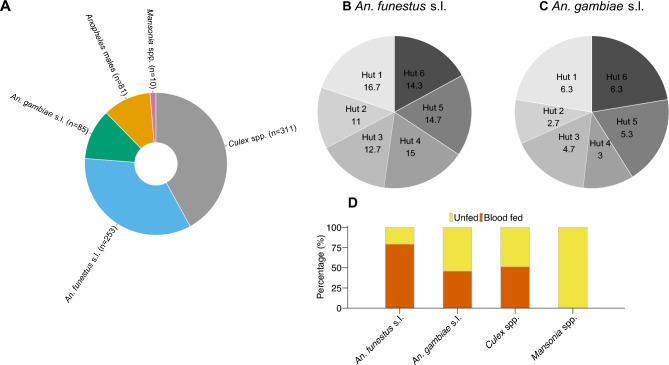
Data from the hut effect test prior to experimental hut trial. (**A**) Vector species composition in Mayuge at the experimental hut site. Relative density of *An. funestus* (**B**) and *An. gambiae* (**C**) mosquitoes collected per hut. (**D**) Blood feeding rate of mosquitoes collected.

During the Hut Effect Test, a total of 740 mosquitoes were collected from the six huts. Out of the 740 mosquitoes, 253 (34.2%) were *An. funestus* females, 85 (11.5%) were *An. gambiae* females, 81 (11.0%) were *Anopheles* males of any species but ignored, 311 (42.0%) were *Culex* species and 10 (3.3%) were *Mansonia* species (Fig. 2A). C*ulex* species were considered in the analysis for being a significant biting nuisance in this area that grows rice which can also transmit lymphatic filariasis in Uganda. There was no significant difference in the number of mosquitoes collected in the huts during the effect test (ANOVA, DF = 17, F-statistic = 0.7112, p = 0.6267) but there was a significant difference in number of mosquitoes collected per week (p = 0.00917). Focusing on the malaria vectors, mosquito average collections per hut were highest with *An. funestus* compared to *An. gambiae* (Fig. 2B and 2C). The blood-feeding rate differed significantly between the malaria vector species (z = 5.9005, p < 0.00001), with the highest rate observed in *An. funestus*, where 79.4% of mosquitoes successfully fed, compared to 45.9% in *An. gambiae* (Fig. 2D). During the hut trial, a total of 3068 mosquitoes were collected. Out of the total collections, 1326 (43.2%) were *An. funestus*, 314 (10.2%) were *An. gambiae*, 731 (23.8%) were *Anopheles* males, 690 (22.5%) were *Culex* species and 7 (0.2%) were *Mansonia* species (Tables 2, 3  and 4). During the trial, there was an overall increase in the mosquito collections as the trial progressed with more mosquitoes collected from Day 21 to Day 36 compared to the earlier days for both *An. funestus* (Fig. 2B) and *An. gambiae* (Fig. 2C).

**Table 2:** Overall results from the performance of LLINs against wild *An. funestus* mosquitoes in experimental huts.

	Control	Interceptor	Interceptor G2	Royal Guard	PermaNet 3.0	Olyset Plus
Total females caught	343	211	177	242	187	166
Deterrence (%)	-	38.5	48.4	29.4	45.5	51.6
Exophily (%)	24.2	29.9	39.5	47.5	46.0	53.0
95% CI	19.67–28.73	23.68–36.03	32.34–46.75	41.23–53.81	38.85–53.13	45.42–60.60
*P-value*		0.14	0.00	0.00	0.00	0.00
Induced exophily (%)	-	NS	20.25	30.77	28.75	38.01
Blood feeding rate (%)	73.2	47.4	39.0	45.9	38.0	23.5
95% CI	68.49–77.87	40.66–54.13	31.80–46.17	39.59–52.15	31.01–44.92	17.04–29.94
*P-value*		0.00	0.00	0.00	0.00	0.00
Blood feeding inhibition (%)	-	35.24	46.73	37.32	48.12	67.89
Personal protection (%)	-	60.16	72.51	55.78	71.71	84.46
Overall mortality (%)	13.4	25.6	70.6	37.6	31.0	36.1
95% CI	9.80–17.02	19.70–31.48	63.91–77.33	31.50–43.71	24.39–37.65	28.84–43.45
*P-value*		0.00	0.00	0.00	0.00	0.00
Corrected mortality (%)	-	14.07	66.07	27.94	20.33	26.25
95% CI	-	9.38–18.76	59.10–73.05	22.29–33.59	14.56–26.10	19.56–32.95
Immediate mortality	6.4	15.6	57.6	28.5	21.9	30.7
Killing effect (%)	-	2	23	13	3	4

**Table 3:** Overall results from the performance of LLINs against wild *An. gambiae* mosquitoes in experimental huts.

	Control	Interceptor	Interceptor G2	Royal Guard	PermaNet 3.0	Olyset Plus
Total Females caught	86	44	38	62	44	40
Deterrence (%)	-	48.8	55.8	27.9	48.8	53.5
Exophily (%)	31.4	43.2	34.2	56.5	52.3	62.5
95% CI	21.59–41.20	28.55–57.82	19.13–49.29	44.11–68.79	37.51–67.03	47.50–77.50
*P-value*		0.18	0.76	0.00	0.02	0.00
Induced exophily (%)	-	NS	NS	36.52	30.43	45.34
Blood feeding rate (%)	62.8	45.5	44.7	21.0	29.5	7.5
95% Conf. limits	52.57–73.01	30.74–60.17	28.93–60.55	10.83–31.10	16.06–43.03	-0.66–15.66
*P-value*		0.06	0.06	0.00	0.00	0.00
Blood feeding inhibition (%)	-	27.61	28.75	66.61	52.95	88.06
Personal protection (%)	-	62.96	68.52	75.93	75.93	94.44
Overall mortality (%)	23.3	25.0	63.2	51.6	61.4	50.0
95% CI	14.33–32.18	12.21–37.79	47.82–78.50	39.17–64.05	46.98–75.75	34.50–65.50
*P-value*		0.83	0.00	0.00	0.00	0.00
Corrected mortality (%)	-	2.27	51.99	36.95	49.66	34.85
95% Conf. limits	-	0–6.68	36.11–67.88	24.94–48.96	34.88–64.43	20.08–49.62
Immediate mortality (%)	16.3	18.2	52.6	41.9	50.0	45.0
Killing effect (%)	-	1	16	26	20	12

**Table 4:** Results from the performance of LLINs against wild *Culex* mosquitoes in experimental huts.

	Control	Interceptor	Interceptor G2	Royal Guard	PermaNet 3.0	Olyset Plus
Total females caught	173	86	104	109	111	107
Deterrence (%)	-	50.3	39.9	37.0	35.8	38.2
Exophily (%)	42.8	61.6	46.2	52.3	58.6	57.9
95% CI	35.40–50.15	51.35–71.91	36.57–55.74	42.92–61.67	49.39–67.72	48.59–67.30
*P-value*		0.00	0.58	0.12	0.01	0.01
Induced exophily (%)	-	32.95	NS	NS	27.58	26.51
Blood feeding rate (%)	37.6	15.1	10.6	11.0	7.2	8.4
95% CI	30.36–44.79	7.55–22.69	4.67–16.49	5.13–16.89	2.40–12.02	3.15–13.67
*P-value*		0.00	0.00	0.00	0.00	0.00
Blood feeding inhibition (%)	-	59.77	71.85	70.70	80.82	77.61
Personal protection (%)	-	80.00	83.08	81.54	87.69	86.15
Overall mortality (%)	22.0	40.7	37.5	27.5	38.7	36.4
95% CI	15.80–28.13	30.31–51.08	28.20–46.80	19.14–35.91	29.68–47.80	27.33–45.57
*P-value*		0.00	0.01	0.29	0.00	0.01
Corrected mortality (%)	-	24.01	19.91	7.12	21.49	18.56
95% CI	-	14.98–33.03	12.23–27.58	2.29–11.95	13.85–29.14	11.19–25.93
Immediate mortality	21.4	36.0	32.7	24.8	34.2	33.6
Killing effect (%)	-	14	17	12	19	17

### Performance of bednets against An. funestus mosquitoes

**Fig. 3:**
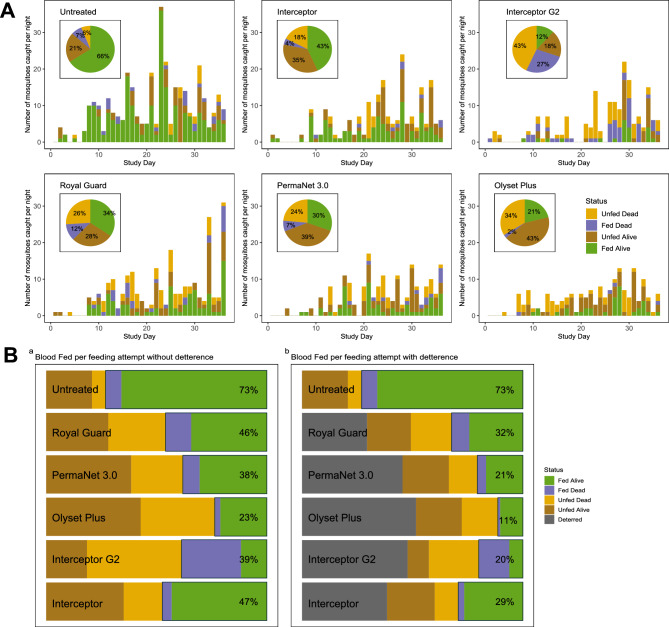
Data from the experimental hut trial, showing nightly variability in mosquito mortality, blood-feeding, and numbers caught for *An. funestus*. (**A**) Breakdown of mosquito numbers in the control arm (untreated net), Interceptor, Interceptor G2, Royal Guard, PermaNet 3.0 and Olyset Plus LLINs over the course of the trial. The height of each bar indicates the total number of mosquitoes entering the hut each night. Bar colour denotes the mosquito mortality at 24 and 72 h and blood-feeding status at 24 h following collection (see the legend in panel A (Olyset Plus) or panel B for the description of the mosquito status). (**B**) Summary measures over the whole trial for the outcome of a single feeding event by a blood-feeding mosquito: being deterred from entering the hut (grey shading, calculated by the difference in the number of mosquitoes caught in the control arm relative to treated nets), mosquitoes being alive and unfed, unfed and dead, fed and dead or successfully blood-fed and alive (green, note the percentage is in the second plot is different from pie charts in panel due to the actions of deterrence).

In the 36-night experimental hut trial, Interceptor G2 emerged as a preeminent mosquito control tool, exhibiting efficacy across multiple metrics (Fig. 3). It achieved the highest overall mortality at 70.6% and a killing effect of 23.0% (Table 2), with 43% of killed mosquitoes being unfed and 27.0% blood fed (Fig. 3A), outperforming the other next-generation bednets; Royal Guard (37.6%), PermaNet 3.0 (31.0%) and Olyset (36.1%), and pyrethroid-only net, Interceptor (25.6%). The highest proportion of successfully blood fed and alive mosquitoes were observed in Interceptor (43.0%) and lowest interceptor G2 (12.0%) (Fig. 3A). Interceptor G2 also excelled in deterrence (48.4%) although this was within similar range as PermaNet 3.0 (45.5%) and Olyset Plus (51.6%) but slightly higher than Interceptor (38.5%) and Royal Guard (29.4%) (Table 2). However, the PBO net, Olyset Plus, proved to be most adept at thwarting blood-feeding, with only 23.5% of mosquitoes being able to blood feed hence inhibiting blood feeding by 67.9%. If we consider deterrence rates, which was highest in Olyset Plus, the blood feeding rate was even lowered further to 11.0% compared to the other nets; Royal Guard (32.0%), PermaNet 3.0 (21.0%) Interceptor (29.0%) and Interceptor G2 (20.0%). Overall, blood feeding rates were significantly lowered by action of deterrence in most of the bednets (Fig. 3B). Subsequently, Olyset plus delivered the highest personal protection (84.46%) compared to PermaNet 3.0 (71.71%) – with a similar protection rate as Interceptor G2 (72.51%), while personal protection was relatively lower in Interceptor (60.16%) and Royal Guard (55.78%) (Table 2). The daily mortality estimates with tight confidence intervals show that interceptor G2 produced less daily variability compared to the other bednets (Supplementary Fig. 1), underscoring its reliability as a control tool for resistant *An. funestus* populations.

### Performance of bednets against An. gambiae mosquitoes

**Fig. 4:**
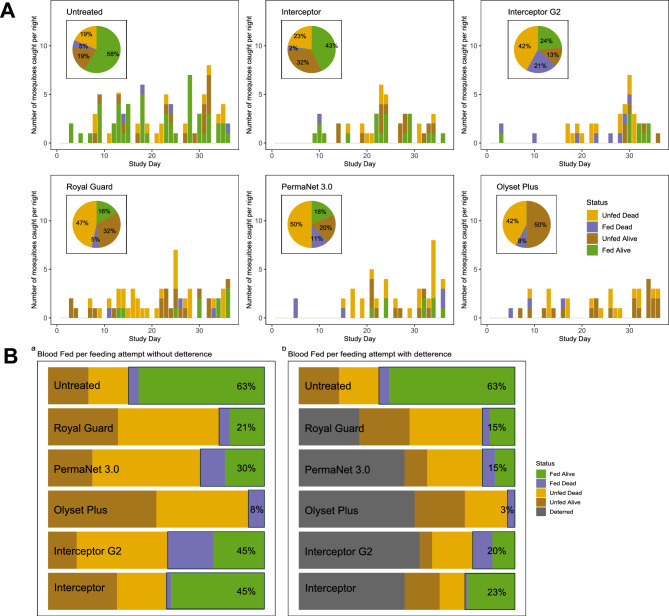
Data from the experimental hut trial, showing nightly variability in mosquito mortality, blood-feeding, and numbers caught for *An. gambiae*. (**A**) Breakdown of mosquito numbers in the control arm (untreated net), Interceptor, Interceptor G2, Royal Guard, PermaNet 3.0 and Olyset Plus LLINs over the course of the trial. The height of each bar indicates the total number of mosquitoes entering the hut each night. Bar colour denotes the mosquito mortality at 24 and 72 h and blood-feeding status at 24 h following collection (see the legend in panel A (Olyset Plus) or panel B for the description of the mosquito status). (**B**) Summary measures over the whole trial for the outcome of a single feeding event by a blood-feeding mosquito: being deterred from entering the hut (grey shading, calculated by the difference in the number of mosquitoes caught in the control arm relative to treated nets), mosquitoes being alive and unfed, unfed and dead, fed and dead or successfully blood-fed and alive (green, note the percentage is in the second plot is different from pie charts in panel due to the actions of deterrence).

Similarly, as reported against *An. funestus* mosquitoes, Interceptor G2 also exhibited high efficacy across multiple metrics against *An. gambiae* mosquitoes (Fig. 4). It equally achieved the highest overall mortality at 63.2% but relatively lower killing effect of 16.0% (Table 3), with 42.0% of killed mosquitoes being unfed and 21.0% blood fed (Fig. 4A). Unlike with *An. funestus*, Interceptor G2 did not outperform other new generational bednets since mortality was within similar range for Royal Guard; 51.6% (39.17–64.05), PermaNet; 61.4% (46.98–75.75) and Olyset; 50.0% (34.50–65.50), but did outperform pyrethroid-only net, Interceptor; 25.0% (12.21–37.79). Similar to findings observed with *An. funestus*, the highest proportion of successfully blood fed and alive mosquitoes were observed in Interceptor (43.0%) but the lowest proportion was with Olyset Plus (0.0%) instead of Interceptor G2 (Fig. 4A). Further contrasts were observed in deterrence rates where except for Royal Guard (27.9%), Interceptor, Interceptor G2, PermaNet 3.0 and Olyset Plus all produced similar rates (within 47–56%). (Table 3). Olyset Plus still significantly proved to be most adept at thwarting blood-feeding, with only 7.5% of mosquitoes being able to blood feed giving a blood feeding inhibition rate of 88.06%. Considering deterrence rates, which was highest in Interceptor G2, the blood feeding rate was lowest in Olyset Plus (3.0%), followed by Royal Guard (15.0%), PermaNet 3.0 (15.0%), Interceptor G2 (20.0%) and Interceptor (23.0%) (Fig. 4B). Overall, blood feeding rates were significantly lowered by action of deterrence in *An. gambiae* compared to *An. funestus* for all the bednets, except Interceptor G2. Again, Olyset Plus delivered the highest personal protection with *An. gambiae* (94.44%) compared to PermaNet 3.0 (75.93%) – with a similar protection rate as Royal Guard (75.93%), while personal protection was relatively lower in Interceptor G2 (68.52%) and Interceptor (62.96%) (Table 3). The daily mortality estimates with tight confidence intervals show that the most of nets produced less daily variability (Supplementary Fig. 2), underscoring its reliability in as a control tool for resistant *An. gambiae* populations.

### Performance of bednets against Culex mosquito species

Against *Culex* mosquitoes, deterrence rate was higher with Interceptor at 50.3%, surpassing next-generation bednets, which clustered around a similar range; Interceptor G2 (39.9%), Royal Guard (37.0%), PermaNet 3.0 (35.8%), and Olyset Plus (38.2%). Exophily rates followed a comparable pattern, but only Interceptor (61.6%), PermaNet 3.0 (58.6%) and Olyset Plus (57.9%) showed statistically significant exit rates compared to the control.

All bednets except Royal Guard yielded significant mortality rates relative to the control. Interceptor had the highest mortality at 40.7% (30.31–51.08), followed closely by Interceptor G2 (37.5%; 28.20 – 46.80), PermaNet 3.0 (38.7%; 26.68–47.80) and Olyset Plus (36.4%; 27.33–45.57). Overall, mortality rates were marginally lower than observed with *An. gambiae* and *An. funestus* malaria vectors.

For personal protection, PBO nets excelled again, with PermaNet 3.0 (87.69%) and Olyset Plus (86.15%) showing the highest rates, though the difference was not significant. Blood-feeding inhibition mirrored this trend. Despite its high mortality, Interceptor recorded the lowest personal protection (80.0%) and blood feeding inhibition (59.77%). Generally, personal protection rates across all bednets were comparable and averaged higher than those in malaria vectors (Table 4).

### Superiority test for mortality, blood feeding and exit rates in Anopheles vectors

**Fig. 5:**
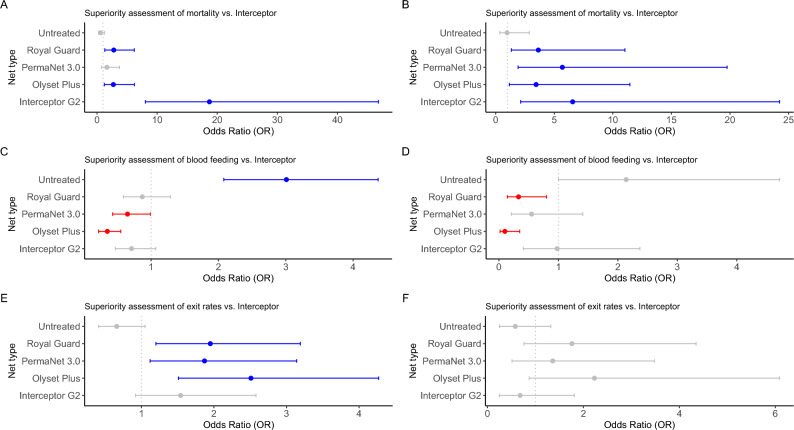
Odds ratio calculation during superiority assessment. The plots show estimates of mosquito mortality after 24 h or 72 (**A, B**), blood feeding rates (**C, D**) and exit rates (**E, F**) in the experimental hut trial (A, C and E are for *An. funestus* while B, D, F, are for *An. gambiae*). The odds ratio graph showing points (with 95% confidence interval estimates on the horizontal lines) of treated LLINs and untreated control net (grey). Overall estimate of the LLIN mortality, blood feeding rates and exit rates is shown in blue if it is above OR = 1.0 or red if it is below OR = 1.0, compared to pyrethroid-only Interceptor net, along with their confidence intervals. Grey dotted horizontal line is OR = 1.0 (cut-off level).

Mosquito mortality rates for *An. funestus* (Fig. 5A, Supplementary Fig. 3A) and *An. gambiae* (Fig. 5B, Supplementary Fig. 3B) revealed that Interceptor G2 is significantly superior to the standard pyrethroid-only net, achieving the highest mortality rates in both populations of highly resistant vectors but with higher superiority in *An. funestus* [OR = 18.7 (8.05–46.8), *P* < 0.001] than *An. gambiae* [OR = 6.57 (2.13–24.24), *P* = 0.002]. Similarly, except for PermaNet 3.0, Royal Guard [OR = 2.79 (1.29–6.2), *P* = 0.01] and Olyset Plus [OR = 2.72 (1.22–6.22), *P* = 0.01] also showed superiority against pyrethroid-only net in resistant *An. funestus* populations, but this was weaker than observed with Interceptor G2. In resistant *An. gambiae* populations, all new-generation bednets demonstrated comparable superiority to Interceptor G2, with PermaNet 3.0 showing the closest performance [OR = 5.69 (1.91–19.76),* P* = 0.003], followed by Olyset Plus [OR = 3.45 (1.16–11.46), *P* = 0.03], and Royal Guard [OR = 3.64 (1.34–11.03), P = 0.014].

Blood feeding rates for *An. funestus* and *An. gambiae* (Fig. 5C & 5D, Supplementary Fig. 3C & 3D) were marginally different. In *An. funestus*, the assessment revealed only PBO nets were significantly superior to the standard pyrethroid-only net, achieving the lowest blood feeding rates for Olyset Plus [OR = 0.35 (0.22–0.55), *P* < 0.001] while PermaNet 3.0 was borderline [OR = 0.65 (0.43–0.99), *P* = 0.0475]. In *An. gambiae*, only Olyset Plus was again significantly superior at levels higher than observed in *An. funestus* [OR = 0.1 (0.02–0.35), *P* < 0.001], as well as Royal Guard [OR = 0.33 (0.14–0.8), *P* = 0.0142]. Despite the superior mortality rate, Interceptor G2 was not superior over standard nets.

In assessing superiority looking at exit rates (Fig. 5E & 5F, Supplementary Fig. 3E & 3F), all the next-generation nets, except for Interceptor G2, were superior to the standard pyrethroid-only net, achieving significant high exit rates in *An. funestus* population [Royal Guard; OR = 1.95 (1.2–3.19), *P* = 0.0068, Olyset Plus; OR = 2.51 (1.51–4.27), *P* = 0.0005 and PermaNet 3.0; OR = 1.87 (1.12–3.14), *P* = 0.0154]. However, no bednet was superior to standard nets in populations of *An. gambiae*.

Generally, in highly resistant populations of *An. funestus* and *An. gambiae*, Interceptor G2 outperformed a standard pyrethroid-only in just one measure of bednet performance. In contrast, Olyset Plus demonstrated superiority in three performance metrics against a standard net in both vectors. For *An. funestus* populations, Royal Guard and PermaNet 3.0 each excelled in two performance metrics. In *An. gambiae* populations, besides Olyset Plus, only Royal Guard surpassed the standard net in two metrics, while PermaNet 3.0 and Interceptor G2 each were superior in only one metric.

### Impact of resistance markers on efficacy of bednets

The impact of resistance or its escalation was assessed using two markers; *4.3 Kb-SV* and *G454A-Cyp9K1* markers point mutations. The efficacy of the three different types of bednets were evaluated using two critical entomological parameters: mortality and blood feeding rates.

**Fig. 6:**
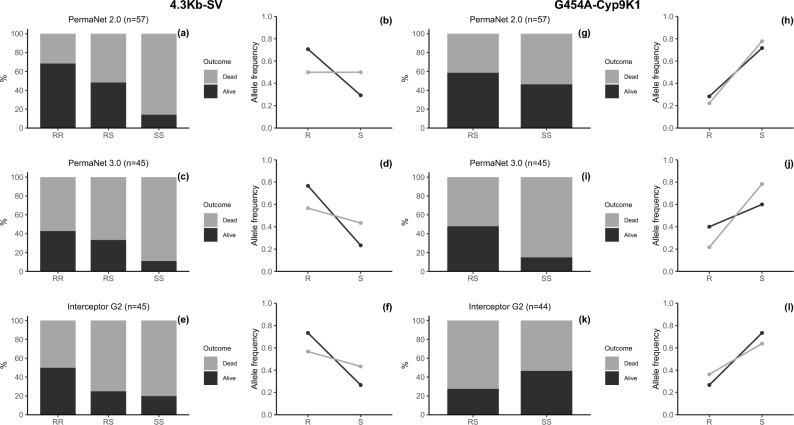
Impact of resistance markers on bednet-induced mortality. Panels (**a, c, e**) and (**g, i, k**) are stacked bars that illustrate the distribution of *4.3 Kb-SV* and *G454A-Cyp9K1* genotypes, respectively, among mosquitoes that either died or survived after exposure to PermaNet 2.0, PermaNet 3.0 and Interceptor G2 LLINs. Panels (**b, d, f**) and (**h, j, l**) are line plots that depict the association between frequency of *4.3 Kb-SV* and *454A-Cyp9K1* R and S alleles, respectively, and the mosquitoes’ ability to survive exposure to these LLINs. The data reveal distinct trends: mosquitoes with RR genotype for *4.3 Kb-SV* and RS genotype for *454A-Cyp9K1* markers exhibit increased survival against PermaNet 2.0 compared to SS genotypes (*4.3 Kb-SV* RR Alive = 68.42% vs. SS Alive = 14.29% and *454A-Cyp9K1* RS Alive = 58.62% vs. SS Alive = 46.43%), with only *4.3 Kb-SV* being significant. For PermaNet 3.0, only the RS genotype of *454A-Cyp9K1* marker is significantly linked to higher survival rates (RS Alive = 48.0% vs. SS Alive = 15.0%). In contrast, RR genotype for *4.3 Kb-SV* is relatively higher in dead than alive mosquitoes exposed to PermaNet 3.0 (Alive = 42.86%, Dead = 57.14%) and balanced in Interceptor G2 (Alive/Dead = 50.0%). The numbers in the brackets are the total number of mosquitoes screened.

The impact of the *4.3 Kb-SV* and *G454A-Cyp9K1* markers were vividly evident. In terms of mortality (Supplementary Fig. 1), there was a significant association between homozygous mutant alleles (RR) for the *4.3 Kb-SV* marker and survival to only PermaNet 2.0 exposure [OR = 13.0 (1.27 – 133.29), P = 0.0221)], with PermaNet 3.0 being marginal. Generally, up to 68.4% of the mosquitoes with RR genotype for *4.3 Kb-SV* marker survived exposure to PermaNet 2.0 compared to 42.9% and 50.0% for PermaNet 3.0 and Interceptor G2, respectively (Fig. 6 a-f). Contrastingly, the *G454A-Cyp9K1* marker was only significantly associated with PermaNet 3.0 survival but in the heterozygote form (RS) since RR genotype was not detected in the hybrids [OR = 5.23 (1.22 – 22.45), P = 0.0235)]. In mosquitoes exposed to PermaNet 3.0, 48.0% having RS genotype for the *G454A-Cyp9K1* marker survived exposure compared to only 15.0% homozygous wild-type genotype (SS), while there was no significant difference in survival with mosquitoes exposed to PermaNet 2.0 and Interceptor G2 (Fig. 6 g-l).

**Fig. 7:**
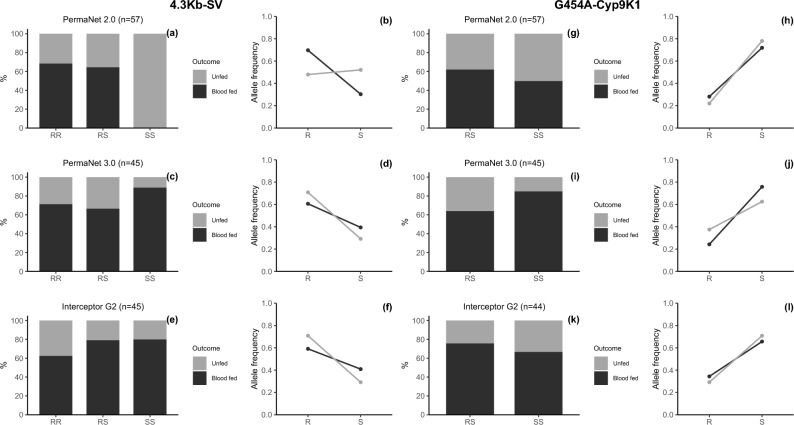
Impact of resistance markers on bednet-induced blood feeding. Panels (**a, c, e**) and (**g, i, k**) are stacked bars that illustrate the distribution of *4.3 Kb-SV* and *G454A-Cyp9K1* genotypes, respectively, among mosquitoes that either blood fed or failed to blood feed after exposure to PermaNet 2.0, PermaNet 3.0 and Interceptor G2 LLINs. Panels (**b, d, f**) and (**h, j, l**) are line plots that depict the association between frequency of *4.3 Kb-SV* and *454A-Cyp9K1* R and S alleles, respectively, and the mosquitoes’ ability to blood feed in the presence of these LLINs. The data reveals RR genotype can increase chances of mosquitoes with *4.3 Kb-SV* to blood feed in presence of the LLINs and this was true and significant only for PermaNet 2.0 (*4.3 Kb-SV* RR Fed = 68.42% vs. SS Fed = 0.0%) but not with *454A-Cyp9K1* marker or other net types. The numbers in the brackets are the total number of mosquitoes screened.

The *4.3 Kb-SV* marker was significantly associated with blood-feeding success on PermaNet 2.0, with both RR (P = 0. 0.00261) and RS (P = 0.0025) genotypes showing higher feeding rates than SS. At the allele level, the resistance allele (R) increased feeding odds [OR = 2.5, 95% CI:1.16–5.41; P = 0.0211] (Supplementary Table 1). Strikingly, no RR genotypes for the *4.3 Kb-SV* marker were detected among unfed mosquitoes exposed to PermaNet 2.0, contrasting with PermaNet 3.0 (*4.3 Kb-SV* Unfed RR = 88.9%) and Interceptor G2 (*4.3 Kb-SV* Unfed RR = 80.0%) – though these differences were non-significant (Fig. 7).

**Fig. 8:**
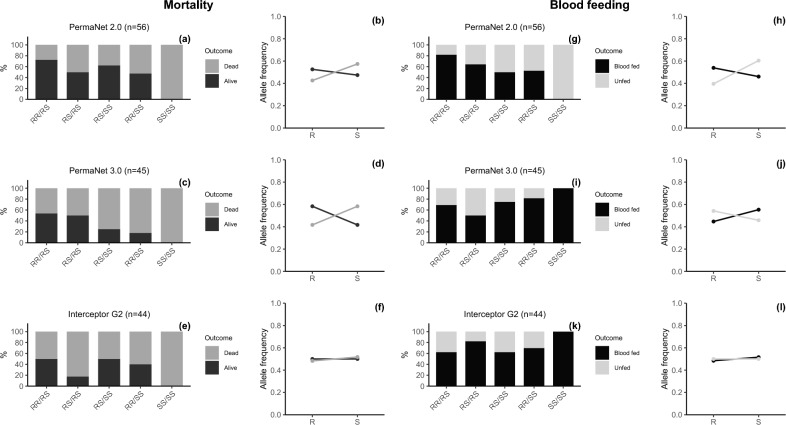
Combined Impact of resistance markers on bednet-induced mortality and blood feeding. Panels (**a, c, e**) and (**g, i, k**) are stacked bars that illustrate the distribution of combined genotypes of both markers, evaluated in terms of mortality and blood feeding success, respectively, after exposure to PermaNet 2.0, PermaNet 3.0 and Interceptor G2 LLINs. Panels (**b, d, f**) and (**h, j, l**) are line plots that depict the association between frequency of R and S alleles from the combination of both markers with mortality and blood feeding success, respectively. The results indicate that mosquitoes with RR/RS genotype combination exhibit a modestly increased likelihood of survival, which was statistically significant when compared to only SS/SS genotypes in mosquitoes exposed to PermaNet 2.0 (RS/RR Alive = 72.7% vs. SS/SS Alive = 0.0%) and PermaNet 3.0 (RS/RR Alive = 53.8 vs. SS/SS Alive = 0.0%). Additionally, this RR/RS genotype combination significantly enhances blood-feeding success against PermaNet 2.0 compared to SS/SS genotypes (RS/RR Fed = 81.8% vs. SS/SS Fed = 0.0%).) and, to a lesser but opposite extent (not significant), for Interceptor G2 (RS/RR Fed = 62.5% vs. SS/SS Fed = 100.0%). The numbers in the brackets are the total number of mosquitoes screened.

The combined effect of both markers showed that possessing RR/RS genotype had an additive advantage to surviving exposure to both PermaNet 2.0 (P = 0.0256) and PermaNet 3.0 (P = 0.05) but not Interceptor G2 (Supplementary Table 2). Generally, the survival rate was higher in individuals that possessed RR/RS, RS/RS, RR/SS genotypes compared to SS/SS genotypes (Supplementary Table 2, Fig. 8 a, b, c, d, e, f). Similarly, individuals possessing RR/RS genotypes had an added advantage to successfully take a blood meal against only PermaNet 2.0 (P = 0.011) and not the other type of bednets (Supplementary Table 3, Fig. 8 g, h, I, j, k, l).

## Discussion

The deployment of long-lasting insecticidal nets (LLINs) represents a cornerstone in malaria control efforts across endemic regions. With over 20 distinct LLIN types currently available from various manufacturers and distributed globally^57^, their effectiveness is increasingly challenged by multiple factors, notably the rapid escalation of insecticide resistance in sub-Saharan Africa. To address this, our study evaluated the efficacy of major prequalified LLINs against highly resistant populations of *An. funestus* and *An. gambiae* in Uganda, aligning with the country’s recent large-scale LLIN distribution efforts. These investigations are critical to understanding the practical impact of next-generation bednets in regions grappling with intense resistance pressures.

### Efficacy of PBO LLINs against highly resistant malaria vectors

This study revealed significant variation in the efficacy of long-lasting insecticidal nets (LLINs) between *An. funestus* and *An. gambiae* populations in Uganda. PermaNet 3.0 and Olyset Plus elicited diminished mortality in *An. funestus* compared to *An. gambiae*. PermaNet 3.0 generally surpassed Olyset Plus in terms of mortality, except for *An. funestus*, where the difference was less pronounced. Conversely, Olyset Plus exhibited marginally superior blood-feeding inhibition in both species, surpassing pyrethroid-only bednets and PermaNet 3.0, likely attributable to permethrin’s potent excito-repellent attributes relative to deltamethrin or alpha-cypermethrin^58^. The observed elevated deterrence and exophily rates with Olyset Plus underscore its repellent prowess against resistant vectors.

Experimental hut trials yielded consistently lower mortality than WHO cone assays^15,24,37^, suggesting that tarsal contact mortality alone cannot encapsulate field-relevant outcomes. Behavioural circumvention in resistant mosquitoes, provoked by PBO nets’ irritancy^59^, likely modulates probing and biting behaviours in natural environs. The reduced PBO nets’ efficacy against *An. funestus* in Uganda, also observed in other geographical settings^5,18^, likely stems from its aggravated pyrethroid resistance status compared to *An. gambiae*^37,60^.

While experimental hut trials offer critical insights, low mortality rates should not be viewed as a definitive measure of poor LLIN performance in community settings. Instead, they highlight the broader impact of insecticide resistance on LLIN efficacy, as demonstrated in previous studies^5,38,61^. Despite the reduced mortality, PBO LLINs conferred substantial personal protection, particularly against *An. gambiae*. Cluster-randomised trials in Uganda and Tanzania corroborated PBO nets’ superiority over pyrethroid-only nets in both clinical and entomological metrics^9,12,13,62^. However, in Uganda, *An. funestus* density was largely unaffected and surged two years post-intervention, contrasting with a decline in *An. gambiae* numbers^35^. This trend mirrors a review by Msugupakulya et al. that showed the ascendancy of *An. funestus* as a dominant vector in eastern and southern Africa from 2010 to 2022 coinciding with large-scale distribution of LLINs^63^.

Our study indicates that PBO LLINs may have limited efficacy in areas dominated by highly resistant *An. funestus* compared to *An. gambiae*-prevalent areas. These outcomes can however be affected by resistance dynamics, underlying mechanisms, and net usage patterns. Although our study did not include PermaNet 2.0 or Olyset 2.0 for direct comparison with PBO nets, we evaluated Interceptor, a pyrethroid-only LLIN. As expected, Interceptor underperformed relative to PBO nets, reinforcing the superior efficacy of PBO nets, and aligning with findings from Tanzania^27^. Notably, Interceptor’s performance appears to have declined since 2006, when mosquito populations were more susceptible to pyrethroids^64^, highlighting the impact of escalating resistance on the effectiveness of pyrethroid-only LLINs.

### Efficacy of recently prequalified new generation dual ai LLINs against highly resistant mosquitoes, with focus on chlorfenapyr-based nets

Our study revealed that Interceptor G2 net achieved superior mortality against both *An. funestus* and *An. gambiae* in Uganda, with marked efficacy against highly resistant *An. funestus*. These findings align with other semi-field trials reporting high mortality for Interceptor G2 against both vectors^24–27^. African malaria vectors are largely susceptible to chlorfenapyr, though resistance was noted in *An. gambiae* from Cameroon, not yet in Uganda by 2021^42^. Interceptor G2 exhibited lower blood-feeding inhibition and personal protection than pyrethroid-only or PBO nets, with greater efficacy against *An. funestus* than *An. gambiae*, likely due to chlorfenapyr negligible excito-repellent properties and alpha-cypermethrin reduced repellence compared to permethrin in Olyset Plus and to deltamethrin in PermaNet 3.0^58^.

Interceptor G2 proved highly effective against resistant *An. funestus*, positioning it as the preferred LLIN in *An. funestus*-dominant regions. Against *An. gambiae*, its performance was comparable to PBO nets, only modestly surpassing pyrethroid-only nets. Cluster-randomised trials in Tanzania and Benin reported high efficacy of Interceptor G2 nets leading to reduced malaria infections^62,65^. Although low-to-moderate pyrethroid resistance was reported in the Tanzanian and Benin studies compared to an area like Mayuge with resistance escalation, the predominance of cytochrome P450-based mechanisms in malaria vectors especially *An. funestus*^66–70^, explains the high efficacy of chlorfenapyr nets in this vector compared to *An. gambiae* where resistance is driven by both metabolic and target-site mechanisms^71,72^. Nonetheless, modelling suggests pyrethroid-pyrrole LLINs could avert 65–75% of malaria cases, varying by epidemiological context^73^. The 2023 Interceptor G2 and PermaNet Dual LLIN distribution in Uganda particularly in high-resistance areas, awaits evaluation.

Our evaluation of Royal Guard, an LLIN combining PPF and pyrethroids, revealed no effect of PPF on oviposition rates (Supplementary Fig. 4) but revealed species-specific performance. Against *An. funestus*, Royal Guard outperformed the pyrethroid-only Interceptor in mortality and exit rates but not in blood-feeding inhibition or personal protection. In *An. gambiae*, it surpassed pyrethroid-only nets in mortality and blood-feeding inhibition, matching PBO nets and exceeding Olyset Plus in personal protection. These results align with a Ugandan trial showing comparable malaria outcomes for Royal Guard and PBO nets in *An. gambiae*-dominant areas^28,30,35^. Royal Guard was more effective against resistant *An. gambiae* than *An. funestus*, surpassing performance in Tanzania^27^, Benin^74^, and Cameroon^24^. These interspecies and geographic disparities likely reflect variations in resistance mechanisms, particularly the overexpression cytochrome P450s, with PPF and pyrethroids metabolised by shared cytochrome P450s^75^. This overlap may lead to synergistic or antagonistic effects across strains, species and regions since resistance mechanisms are geographically distinct^72,76,77^.

### Impact of resistance escalation on performance of bednets

Numerous studies have explored the impact of insecticide resistance on bednet efficacy. Currently, the primary approach involves using diagnostic resistance markers associated with resistance genes as proxies to assess this impact. However, recent advancements in whole-genome sequencing^78^ are paving the way for more comprehensive methods to investigate resistance mechanisms and their effects on vector control tools.

In this study, both the *4.3 Kb-SV* and *G454A-CYP9K1* markers showed an association with survival to pyrethroid-only and PBO bednet respectively. Only the *4.3 Kb-SV* marker showed a significant association with blood feeding. In Cameroon where both markers have spread to and were evaluated for the first time, similar results were obtained with *4.3 Kb-SV* significantly associating with pyrethroid-only bednets^52^ and *G454A-CYP9K1* associating with survival to only PBO nets but not Royal sentry (an alpha-cypermethrin-only) bednet in experimental hut trials^51^. Furthermore, we show for the first time that the combination of both markers significantly enhances mosquitoes’ ability to survive both pyrethroid-only and PBO nets but not chlorfenapyr nets, highlighting the impact of resistance escalation on vector control tools.

The *4.3 Kb-SV* marker, first identified in eastern Uganda in 2014 and already fixed by that time^52^, was likely under strong selection from earlier interventions like standard LLINs and IRS between 2003 and 2014^79,80^. This historical context may explain the absence of a clear association with survival hence cross-resistance with the newer bednets introduced post-2014. Furthermore, and consistent with prior studies^5,38,50,61,81^, we observed a significant association between the *4.3 Kb-SV* marker and blood-feeding rates, suggesting a potential connection to probing and biting behaviours, a critical adaptation influencing vectorial capacity. In contrast, the selection of the *G454A-Cyp9K1* marker was likely exacerbated by the distribution of next-generational PBO bednets compared to pyrethroid-only bednets, given its increase in frequency post 2014^51^, reinforcing the role of metabolic resistance in diminishing the efficacy of bednets.

We show for the first time that when both markers are present, their combined effect significantly boosts mosquito survival against PBO and pyrethroid-only bednets, with a more pronounced impact on pyrethroid-only nets. This synergy likely reflects complementary resistance mechanisms responding to mixed selection pressures, explaining the persistence of *An. funestus* populations despite deployment of earlier next-generation bednets^63^. Our results indicate that the molecular basis of these resistance mechanisms further elucidates the impact of resistance escalation to vector control. For instance, the *4.3 Kb-SV* marker is associated with the overexpression of CYP6P9A and CYP6P9b genes^52^, which have been linked to intense pyrethroid resistance^36,37^. Similarly, the CYP9K1 gene has also been shown to be involved in resistance escalation^36,37^ and the *G454A-Cyp9K1* marker confers this resistance by accelerating the metabolism of pyrethroids, rather than increasing enzyme overexpression^51^.

The absence of a clear impact of both markers on Interceptor G2 bednet indicates that they are specific to pyrethroid resistance, with no impact on the chlorfenapyr which has a distinct mode of action. Chlorfenapyr distinct mode action likely masks the killing effect of pyrethroids in the dual ai Interceptor G2 and PermaNet Dual nets. In *An. funestus*, resistance is predominantly driven by overexpression of cytochrome P450 enzymes^66,67,69,70,76,82–84^, which are induced by pyrethroids that may in turn activate chlorfenapyr, thereby enhancing its toxicity. Consequently, pyrethroid resistance escalation may not compromise Interceptor G2 and PermaNet Dual nets, explaining the high performance of these nets against *An. funestus* populations in this study.

### Efficacy LLINs against Culex mosquitoes

Our study also assessed the performance of LLINs against Culex species in Uganda, a vector often overlooked in experimental hut trials due to its limited role in major vector-borne disease transmission in sub-Saharan Africa. Mortality rates were low, like those observed for *An. funestus*, except for Interceptor G2, which exhibited higher efficacy. Most LLINs provided robust personal protection against *Culex* species, consistent with findings from Tanzania^15^. This highlights the efficacy of insecticide-treated nets (ITNs) in reducing *Culex* biting nuisance and potential pathogen transmission, including Japanese encephalitis (virus), lymphatic filariasis (parasite) and West Nile fever (virus)^85^. While viral disease risk is minimal in Uganda, *Culex* mosquitoes alongside *Anopheles*, may contribute to lymphatic filariasis transmission in Northern Uganda and Lake Kyoga basin^86,87^. However, mass drug administration effectively controls lymphatic filariasis^88^, rendering *Culex* mosquitoes of minor public health significance in Uganda. Continued surveillance remains essential in lymphatic filariasis-endemic regions to address potential emerging risks.

### Limitations

The study had a few limitations. Firstly, in the *An. gambiae* data, the total number of mosquito collections were relatively low for the treatment arms (ranging from 1.1 to 1.7 mosquitoes per night). We did not break down the species composition of *An. gambiae* s.l. since resistance profiles and behaviour can be significantly different. Therefore, the *An. gambiae* data should be interpreted with caution. Secondly, in the genetic crossing experiment, the use of hybrid mosquitoes can have some genetic variability leading to unpredictable phenotypic plasticity such as altered fitness affecting survival and recapture rates, changes in probing and biting behaviours, altered genetic diversity affecting resistance-associated genes^48–50,89^. However, our genotype–phenotype results align closely with prior studies involving free-flying mosquitoes^51,52^, further confirming the validity of our findings. Thirdly, the experimental design of the study did not assess the impact of net washing which might not accurately reflect real-world conditions where nets are washed and used over an extended period. Evaluating LLINs in both unwashed and washed states is recommended by WHO guidelines and would have provided a more comprehensive understanding of their efficacy and durability in practical use. However, this would not have affected the final goal of the study, which was to examine the performance of new bednets, as washing typically diminishes the insecticidal effectiveness. Lastly, another limitation of this study is that Interceptor, the pyrethroid-only comparator, differs from PBO and PPF nets in both fabric type and pyrethroid component, making it a suboptimal comparator according to WHO guidelines for superiority trials. Consequently, the reported superiority should be interpreted with caution, although these differences are unlikely to have materially affected the outcomes, as previous experimental hut trials have shown little or no difference in performance among pyrethroid-only nets against resistant vector populations^90^.

## Conclusion

Insecticide resistance has profoundly undermined the efficacy of LLINs in sub-Saharan Africa, with Uganda standing as a compelling case study. Our investigation unveiled striking disparities in LLIN performance across two major vector species, with Interceptor G2 net demonstrating superior efficacy especially in *An. funestus*, albeit showing reduced performance in *An. gambiae*. In contrast, PBO LLINs and Royal Guard exhibited commendable performance against *An. gambiae* but not *An. funestus*. The heterogeneity in LLIN performance underscores the urgency for precise vector mapping and a nuanced, possibly rotational, LLIN deployment strategy. Moreover, our findings reveal that resistance mechanisms in *An. funestus* significantly compromise the performance of pyrethroid-only and PBO nets, while Interceptor G2 net remain largely unaffected. Hence, novel resistance mechanisms could emerge from the selection pressure to counter chlorfenapyr-treated nets given their recent nationwide rollout in Uganda in 2023, necessitating vigilant resistance surveillance. Ultimately, these insights reinforce the imperative for adaptive, evidence-informed vector control strategies to safeguard the long-term gains in malaria control amidst an evolving insecticide resistance landscape.

## Supplementary Information

Supplementary Information.

## Data Availability

All datasets generated or analysed during this study are included in this published article and its supplementary files.
