# Supplementary material for: Chlorfenapyr bednets effectively overcome pyrethroid resistance escalation in highly resistant Anopheles malaria vectors in Uganda
Source: Sci Rep. 2026 Jan 2;16:4292. doi: 10.1038/s41598-025-34493-3 (PMC12858971; doi:10.1038/s41598-025-34493-3)
Supplement: Supplementary file 1 — Supplementary Information. [file 41598_2025_34493_MOESM1_ESM.pdf]

Supplementary material

Supplementary Figure 1: *An. funestus* daily mortality estimates (and 95% confidence intervals, vertical line) for LLINs, indicating the wide variation observed over the course of the trial. The larger circles indicate mortality estimates with narrower confidence intervals (i.e. higher numbers of mosquitoes present). The colour of the points indicates the hut number.

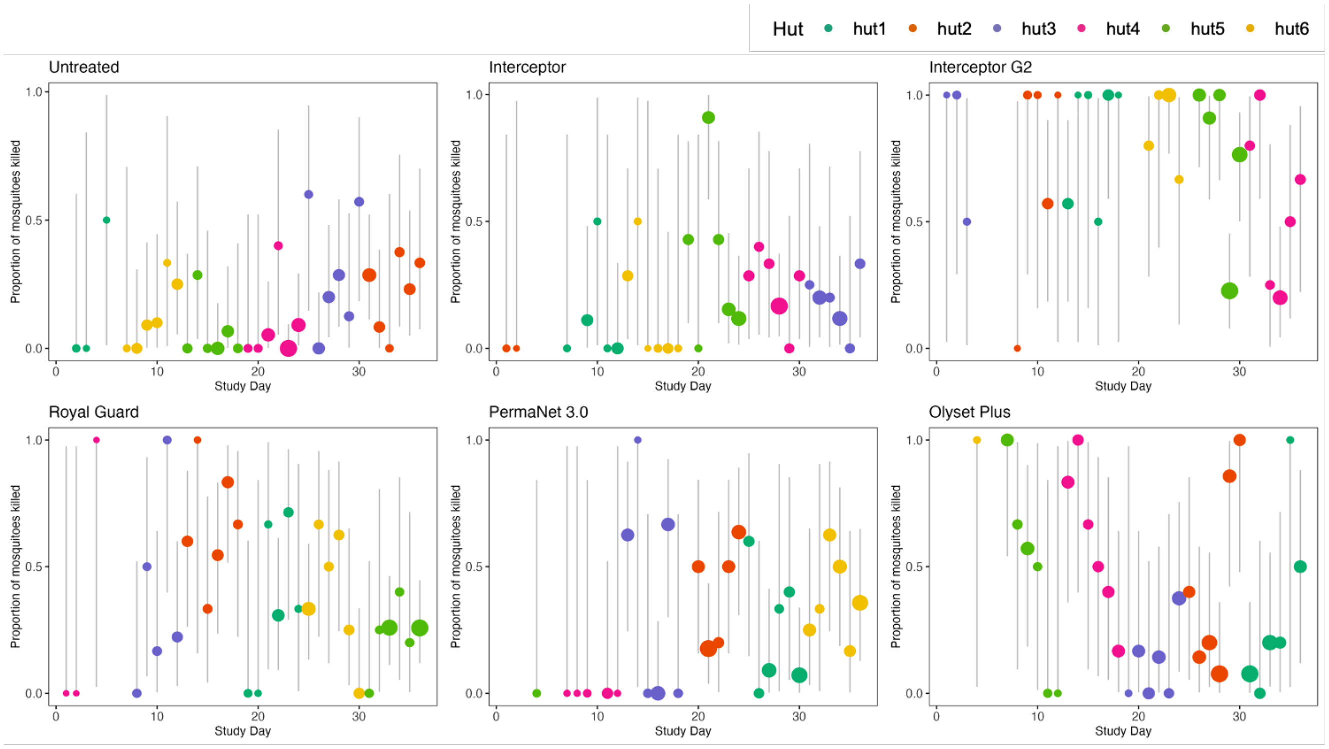

Supplementary Figure 2: *An. gambiae* daily mortality estimates (and 95% confidence intervals, vertical line) for LLINs, indicating the wide variation observed over the course of the trial. The larger circles indicate mortality estimates with narrower confidence intervals (i.e. higher numbers of mosquitoes present). The colour of the points indicates the hut number.

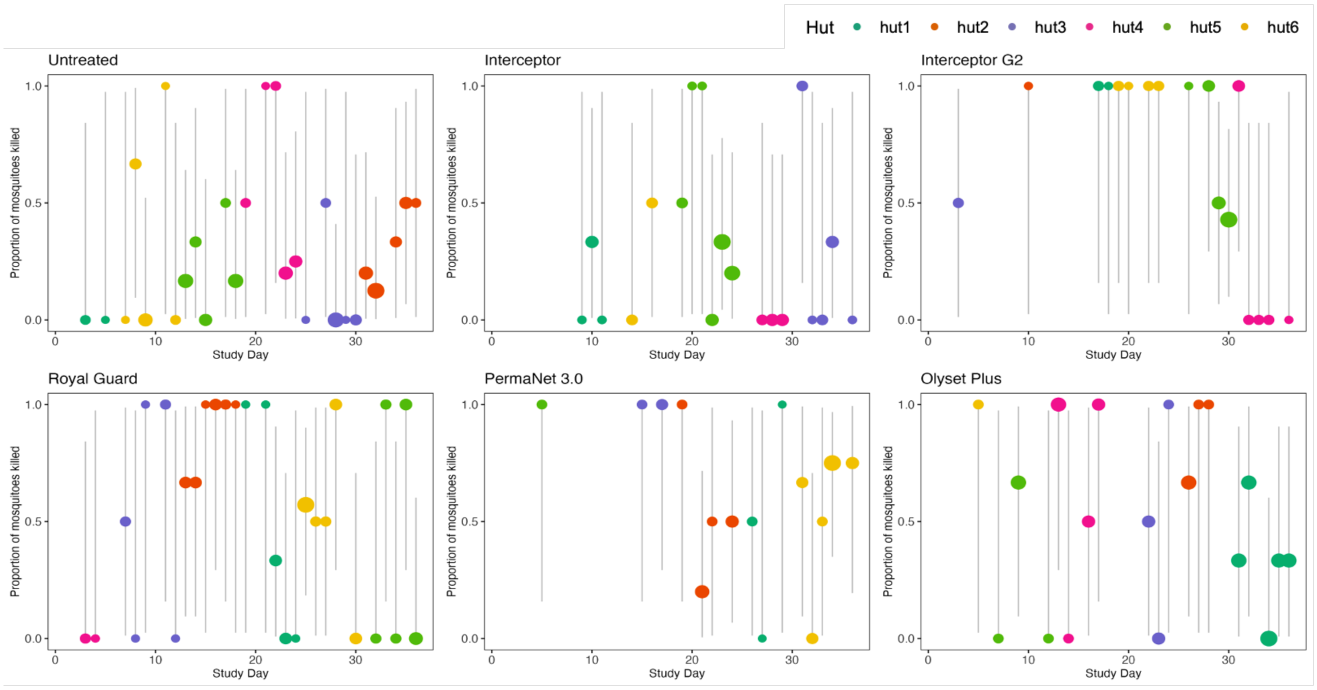

Supplementary Figure 3: Superiority assessment. The bar plot shows the charts of actual and modelled rates (as points in the error bars of bar charts).

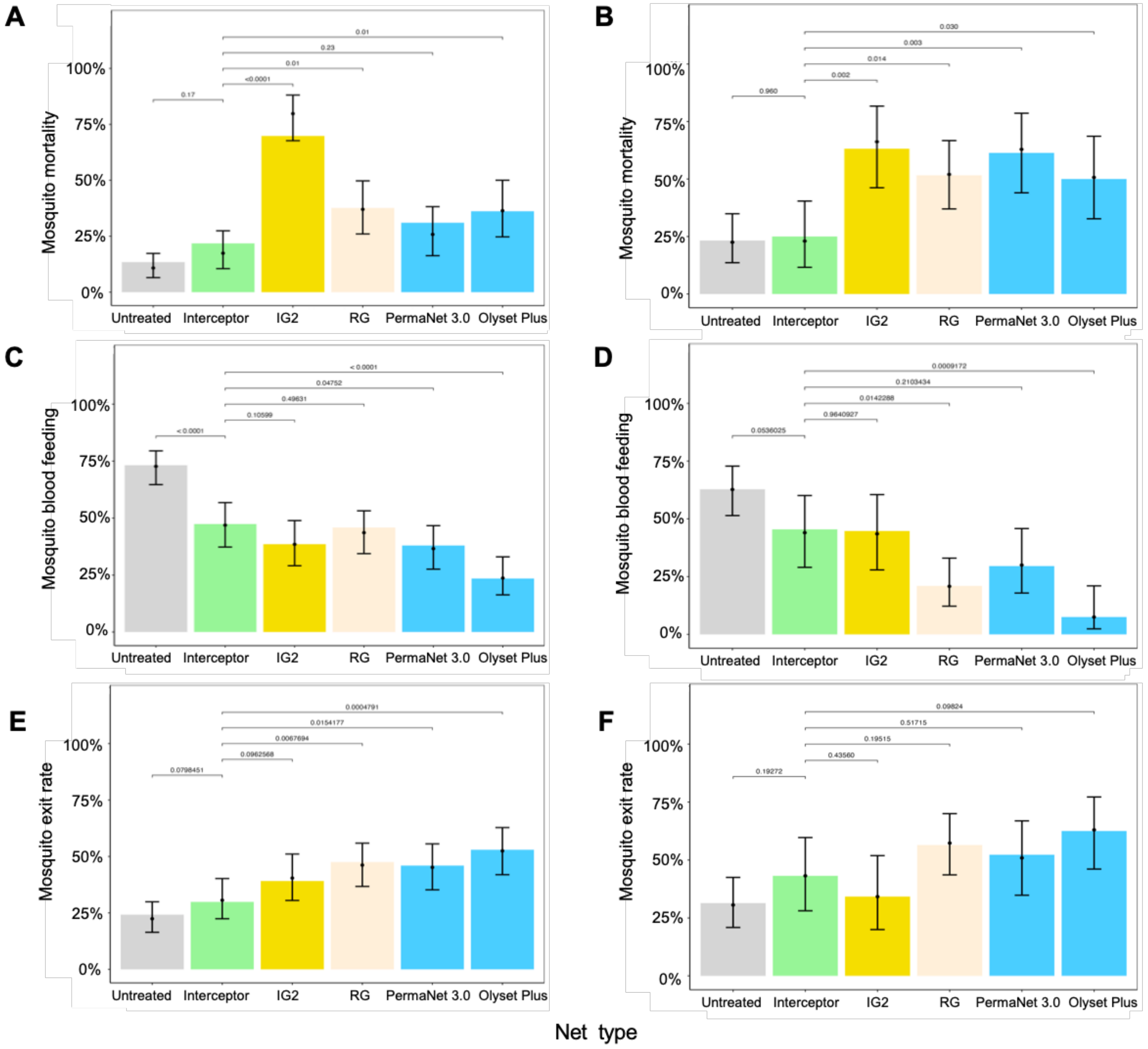

Supplementary Figure 4. Oviposition rates of gravid and blood fed malaria vectors collected from Royal Guard treatment arm**.** A violin plot with the proportion of oviposited mosquitoes showing no difference in oviposition rates of *An. funestus* and *An. gambiae* female mosquitoes collected from Royal Guard huts compared to their controls measured using Bonferroni p-adjusted t-tests with pooled SD method in R software.

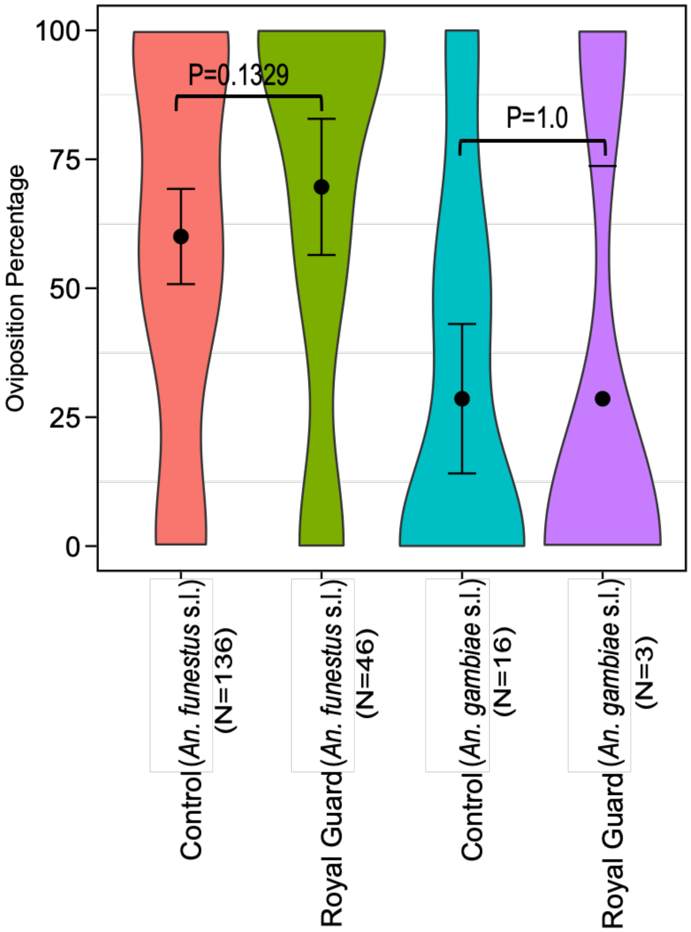

Supplementary Table 1. Correlation between the homozygous or heterozygous genotypes of *4.3Kb-SV* and*G454A-Cyp9K1* verses other genotypes with mortality and blood feeding rates against three types of insecticide-treated nets in experimental huts.

		4.3Kb-SV			***G454A-Cyp9K1***		
		OR	95% CI	P value	OR	95% CI	P value
Mortality (All samples)							
PermaNet 2.0	RR vs SS	13	1.27 - 133.29	0.0221	NA	NA	1
	RR vs RS	2.31	0.7 - 7.65	0.1828	NA	NA	1
	RS vs SS	5.63	0.6 - 52.37	0.1216	1.64	0.57 - 4.66	0.3736
	R vs S	2.41	1.12 - 5.21	0.0260	1.38	0.59 - 3.25	0.4656
PermaNet 3.0	RR vs SS	6.0	0.63 - 57.0	0.1115	NA	NA	1
	RR vs RS	1.5	0.38 - 5.95	0.5885	NA	NA	1
	RS vs SS	4.0	0.39 - 41.51	0.2752	5.23	1.22 - 22.45	0.0235
	R vs S	2.5	0.94 – 6.75	0.0672	2.41	0.93 - 6.26	0.0784
Interceptor G2	RR vs SS	4	0.36 - 44.11	0.2968	NA	NA	1
	RR vs RS	3	0.78 - 11.54	0.1247	NA	NA	1
	RS vs SS	1.3	0.12 - 14.38	0.8747	0.44	0.12 - 1.60	0.2316
	R vs S	2.10	0.81 - 5.48	0.1315	0.64	0.24 - 1.70	0.3816
Blood feeding (All samples)							
PermaNet 2.0	RR vs SS	Inf.	NA	0.00261	NA	NA	1
	RR vs RS	1.19	0.35 - 4.02	0.7928	NA	NA	1
	RS vs SS	Inf.	NA	0.0025	1.64	0.57 - 4.70	0.3756
	R vs S	2.5	1.16 - 5.41	0.0211	1.39	0.59 - 3.29	0.4682
PermaNet 3.0	RR vs SS	0.31	0.03 - 3.07	0.3541	NA	NA	1
	RR vs RS	1.25	0.3 - 5.23	0.7707	NA	NA	1
	RS vs SS	0.25	0.02 - 2.59	0.2752	0.31	0.07 - 1.37	0.1307
	R vs S	0.63	0.23 - 1.74	0.3893	0.53	0.20 - 1.45	0.2323
Interceptor G2	RR vs SS	0.42	0.04 - 4.66	0.5411	NA	NA	1
	RR vs RS	0.44	0.11 - 1.80	0.2778	NA	NA	1
	RS vs SS	0.95	0.09 - 10.5	0.9859	1.57	0.40 - 6.18	0.5365
	R vs S	0.60	0.22 - 1.63	0.3242	1.27	0.46 - 3.53	0.6617

Inf. = Infinity, NA = Not Applicable, OR = Odds Ratio, CI = Confidence Interval.

Supplementary Table 2. Correlation between the combined homozygous or heterozygous genotypes of *4.3Kb-SV* and*G454A-Cyp9K1* verses other genotypes with mortality rates against three types of insecticide-treated nets in experimental huts.

		OR	95% CI	P value
PermaNet 2.0	RR/RS vs RS/RS	2.67	0.49 - 14.46	0.2884
	RR/RS vs RR/SS	1.6	0.23 - 11.27	0.6718
	RR/RS vs RS/SS	2.96	0.6 - 14.73	0.2064
	RR/RS vs SS/SS	Inf.	NA	0.0256
PermaNet 3.0	RR/RS vs RS/RS	1.17	0.2 - 6.8	0.8762
	RR/RS vs RR/SS	3.5	0.5 - 24.27	0.2384
	RR/RS vs RS/SS	5.25	0.8 - 34.43	0.0949
	RR/RS vs SS/SS	Inf.	NA	0.05
Interceptor G2	RR/RS vs RS/RS	4.67	0.72 - 30.11	0.1327
	RR/RS vs RR/SS	1	0.14 - 7.1	1
	RR/RS vs RS/SS	1.5	0.23 - 9.8	0.7044
	RR/RS vs SS/SS	Inf.	NA	0.5556

Inf. = Infinity, NA = Not Applicable, OR = Odds Ratio, CI = Confidence Interval.

Supplementary Table 3. Correlation between the combined homozygous or heterozygous genotypes of *4.3Kb-SV* and*G454A-Cyp9K1* verses other genotypes with blood feeding rates against three types of insecticide-treated nets in experimental huts.

		OR	95% CI	P value
PermaNet 2.0	RR/RS vs RS/RS	2.5	0.38 - 16.42	0.3808
	RR/RS vs RR/SS	4.5	0.57 - 35.52	0.1894
	RR/RS vs RS/SS	4.05	0.68 - 23.95	0.133
	RR/RS vs SS/SS	Inf.	NA	0.011
PermaNet 3.0	RR/RS vs RS/RS	2.25	0.37 - 13.87	0.4259
	RR/RS vs RR/SS	0.75	0.1 - 5.47	0.8117
	RR/RS vs RS/SS	0.5	0.07 - 3.45	0.528
	RR/RS vs SS/SS	0	NA	0.2337
Interceptor G2	RR/RS vs RS/RS	0.36	0.05 - 2.38	0.3336
	RR/RS vs RR/SS	1	0.13 - 7.57	1
	RR/RS vs RS/SS	0.71	0.1 - 5.12	0.7647
	RR/RS vs SS/SS	0	NA	0.6667

Inf. = Infinity, NA = Not Applicable, OR = Odds Ratio, CI = Confidence Interval.
